# Supplementary material for: The Proposed Neurotoxin β-N-Methylamino-l-Alanine (BMAA) Is Taken up through Amino-Acid Transport Systems in the Cyanobacterium Anabaena PCC 7120
Source: Toxins (Basel). 2020 Aug 13;12(8):518. doi: 10.3390/toxins12080518 (PMC7472364; doi:10.3390/toxins12080518)
Supplement: Supplementary file 1 [file toxins-12-00518-s001.pdf]

Supplementary Materials: The Proposed Neurotoxin β-*N*-Methylamino-L-Alanine (BMAA) Is Taken up through Amino-Acid Transport Systems in the Cyanobacterium *Anabaena* PCC 7120

Zi-Qian Wang, Suqin Wang, Ju-Yuan Zhang, Gui-Ming Lin, Nanqin Gan, Lirong Song, Xiaoli Zeng and Cheng-Cai Zhang

Table S1. Amino acid transporters in *Anabaena* sp. PCC 7120 summarized according to previous publications [1,2].

Transporter	ORF (Gene)	Predicted Gene Production	Transported Amino Acids
N-I [3]	*all1046* (*natA*)<br>*alr1834* (*natB*)<br>*all1047* (*natC*)<br>*all1284* (*natD*)<br>*all2912* (*natE*)	ATPase<br>PSB<br>transmembrane protein<br>transmembrane protein<br>ATPase	Pro, Phe, Leu, Gly, Thr, Ala, Ser, Met, Asn, His, Orn, Gln, Glu
N-II [1]	*alr4164* (*natF*)<br>*alr4165* (*natG*)<br>*alr4166* (*natH*)<br>*alr4167* (*bgtA*)	PSB<br>transmembrane protein<br>transmembrane protein<br>ATPase	Asp, Glu, Asn, Gln, Met, Thr, Ala, Ser, Gly, His
Bgt [1]	*alr4167* (*bgtA*)<br>*alr3187* (*bgtB*)	ATPase<br>PSB and transmembrane protein	Lys, Arg, Orn, His, Gln
N-III [2]	*alr2535* (*natI*)<br>*alr2536* (*natJ*)<br>*alr2538* (*natK*)<br>*alr2539* (*natL*)<br>*alr2541* (*natM*)	PSB<br>transmembrane protein<br>transmembrane protein<br>ATPase<br>ATPase	Gly, Pro, Glu, Phe, Leu, Ala, Gln

PSB: periplasmic substrate-binding protein; The order of the presented amino acids for each transporter reflects the contribution of the corresponding transporter to the total uptake of the indicated amino acids by nitrate-grown *Anabaena* filaments, as reported [1,2].

Table S2. Mutations mapped in different BMAA^r mutants.

BMAA^r Strains	ORF Affected	
	alr4167(bgtA)	***all1284(natD)***
M1	deletion of A354	A716G (Tyr 239 Cys)
M2	C253T (R85 stop codon)	C80T (Thr 27 Ile)
M3	-	deletion of A652
M4	-	-
M5	-	A716G (Tyr 239 Cys)
M6	-	-
M7	-	-

M8	T8C (Met 3 Thr)	-
M9	C253T (R85 stop codon)	insertion of TGG203
M10	C253T (R85 stop codon)	-
M11	G263A (Gly 88 Glu)	deletion of A652
M12	-	deletion of A652
M13	insertion of GG127	-
M14	-	-
M15	C217T (Leu 73 Phe)	deletion of A652
M16	-	deletion of A649

-: no mutation detected in the corresponding ORF.

Table S3. Strains and major plasmids.

Strain or Plasmid	Description	Source
**Strains**		
Anabaena sp. PCC7120	Wild type	Pasteur Culture collection
Anabaena ΔnatA	Nm^r; A neomycin-resistance cassette inserted into position 121-720 within the ORF *all1046/natA*	This study
Anabaena ΔnatD	A markerless mutant by removing an internal fragment from 6 to 865 within the ORF *all1284/natD*.	This study
Anabaena ΔnatG	A markerless mutant by removing an internal fragment from 46 to 897 within the ORF *alr4165/natG*	This study
Anabaena ΔbgtA	Nm^r; A neomycin-resistance cassette inserted into position 262 to 696 of the ORF *alr4167/natG*	This study
Anabaena ΔbgtB	Nm^r; A neomycin-resistance cassette inserted into position 265 to 1041 within the ORF *alr3187/bgtB*	This study
Anabaena ΔnatI	A markerless mutant by removing an internal fragment from position 46 to 762 of the ORF *alr2535/natI*	This study
Anabaena ΔnatAΔbgtA	Nm^r Sp^r Sm^r; a construct similar as for *ΔbgtA* but bearing a spectinomycin-resistance cassette was transferred into the Δ*natA* mutant	This study
**Plasmids**		
pCint2	*sacB*-bearing cloning vector	Zhang et al., 2018 [4]
pCint2-Mall1046	Km^r Nm^r; for construction of Δ*natA* by homologous recombination as indicated in (Supplementary Figure. S1)	This study
pCint2-Malr4167	Km^r Nm^r; for construction of Δ*bgtA* (Supplementary Figure. S1)	This study
pCint2-Malr4167-sp	Sp^r Sm^r; for construction of Δ*bgtA* (Supplementary Figure. S1)	This study
pCint2-Malr3187	Km^r Nm^r; for construction of Δ*bgtB* (Supplementary Figure. S1)	This study
pCpf1	Km^r Nm^r; vector carrying the Cpf1 genome editing system	This study [5]
pCpf1-Malr4165R126	Km^r Nm^r; for constructing Δ*natG* by Cpf1 genome editing system (Supplementary Figure. S1)	This study

pCpf1-Malr2535R226	Km^r Nm^r; for constructing Δ*natI* by Cpf1 genome editing system (Supplementary Figure. S1)	This study
pCpf1-Mall1284F610	Sp^r Sm^r; for constructing Δ*natD* by Cpf1 genome editing system Supplementary Figure. S1)	This study

Table S4. Primers used in this study (sequences in minuscule correspond to the overlapping homologous parts in PCR fragments for ligation during cloning, and the sequence in capital correspond to sequences used for DNA amplification during PCR).

Primer	Sequence (5′ to 3′)
PtstspF	AGCACTAGCGTCGGTAGCGCT
Pgfp_spR	GGGAGTACTGATGATCCGGT
PV_14	GCAATGGCAACAACGTTGCG
PV_13	GATCTAGATATCGAATTTCTGCCA
Pall1046F1256m	gcagaaattcgatatctagatcAAGTCAGTCGCGTCGAGA
Pall1046R120	agcgctaccgacgctagtgctAGTGATACTGCCTTGTGCAAC
Pall1046F721	accggatcatcagtactcccGATGGAACTCCAGCCGAA
Pall1046R2119	cgcaacgttgttgccattgcGCACAAACAGCACTGAAGG
Palr4167F1081m	gcagaaattcgatatctagatcGTTGTGGCTAGTACAAGGGA
Palr4167R261	agcgctaccgacgctagtgctTACTTCTCGTCGGATTGC
Palr4167F697	accggatcatcagtactcccTCTTCACCAAACCCCAAGA
Palr4167R2200	cgcaacgttgttgccattgcGGCGAAATCGCGCAACTT
Palr3187F1030m	agaaattcgatatctagatcGACAGGCTTTAGCCGAATAG
Palr3187R264	gcgctaccgacgctagtgctACTTTGCAAGGCGGGGAT
Palr3187F1042	accggatcatcagtactcccGCACTTGCTCAACAACTT
Palr3187R2328	cgcaacgttgttgccattgcTTGTTCAGCGATCGCTCT
Palr4165F1188m	gcagaaattcgatatctagatctGCCACGACTTTATGGTATTC
Palr4165R45	CCAGAAGCGATTATCACG
Palr4165F898	ggcgtgataatcgcttctggAATCGCACCGTACAGATT
Palr4165R2078	caacgttgttgccattgcggatccAAGCAAGGGACATTGAGT
cr_alr4165R126F	agatCAAATTGCGGTTAAGATTACCT
cr_alr4165R126R	agacAGGTAATCTTAACCGCAATTTG
Palr2535F1270m	gcagaaattcgatatctagatctGCAACAGTCCCATCATCT
Palr2535R45	ACCAATTTCTGGTATGCCT
Palr2535F763	aggcataccagaaattggtATGGAAAAGGATGGTAGTCAA
Palr2535R1849	aacgttgttgccattgcggatccTGGCTTTGTACCTCAGAC
cr_alr2535R226F	agatCTAATTCCAGCCCTTGTTCCTT
cr_alr2535R226R	agacAAGGAACAAGGGCTGGAATTAG
Pall1284F1359m	gcagaaattcgatatctagatctCCAGTGTCCCAGACTGTA
Pall1284R6	GTCCATATTGCTAATTAAATAAGCTAAT
Pall1284F865	taattagcaatatggacTGAGCAACACCATTCCACTTA
Pall1284R2168	aacgttgttgccattgcggatccATCGGGGTGAGTGCTATG
cr_all1284F610F	agatGGTGGCAGTATGTATGGCTTAA
cr_all1284F610R	agacTTAAGCCATACATACTGCCACC
Pall1284R924	TTCACCCAAGCCCCAGAC
Palr4167R841	CTCTGTTATCTCCAAGTTACG
P281F	CGATTAGTTCCATCACCATC
P982R	CTACGTAAGCTCTAGCGAATA

P190R	CCCACTACTGCCACTATCATCGA

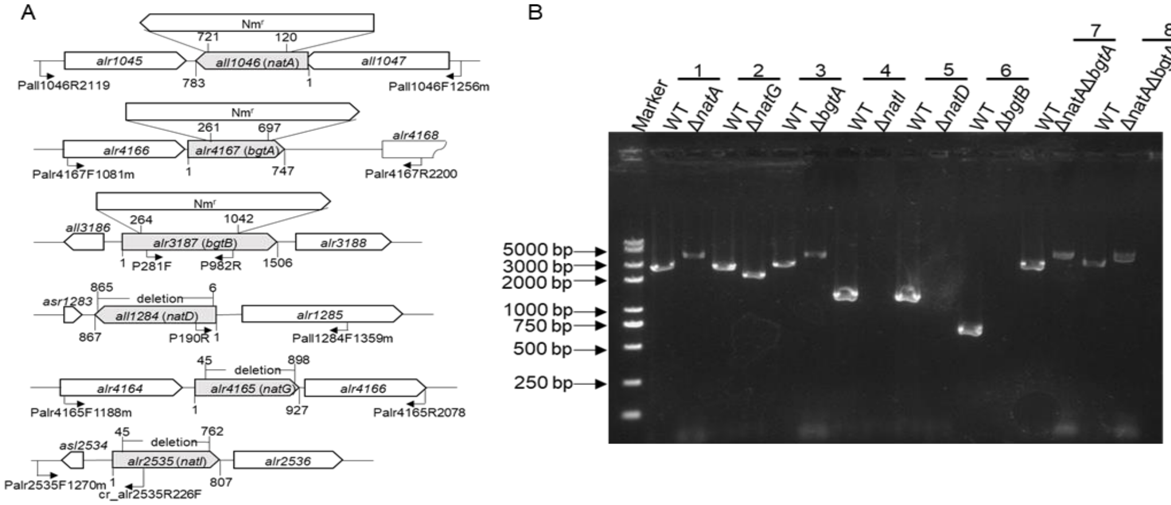

Figure S1. (**A**) Strategy for generating targeted inactivation of genes involved in amino acid transport in *Anabeana*. Nm^r, a gene cassette encoding a resistance marker to the antibiotic neomycin. Each ORF (open-reading frame) was numbered from the beginning at the 5′ end (position 1), till the end at the 3′. *natA*, *bgtA*, and *bgtB* were inactivated by homologous recombination, by replacing an internal gene fragment at positions as indicated by the Nm^r cassette. The other three genes were inactivated by deleting an internal fragment as indicated by using the recently developed technique based on Cpf1 [5,6]. (**B**) Segregation of the mutants determined by PCR. One pair of oligonucleotides, whose sequences as listed in Table S4, were used to check the inactivation of the genes in comparison with the wild type (WT). The relative positions of the primers are indicated in A. In 1 and 7, primers Pall1046F1256m and Pall1046R2119 are used, and the fragment from the mutants is 4603 bp as compared to 3647 bp in WT. In 2, primers Palr4165F1188m and Palr4165R2078 are used, generating a fragment of 2460 bp from *ΔnatG* and a fragment of 3313 bp from WT. In 3 and 8, a fragment of 4689 bp can be amplified from *ΔbgtA* and *ΔnatAΔbgtA* and a shorter fragment of 3320 bp from WT, using the primers Palr4167F1081m and Palr4167R2200. In 4, by using primers Palr2535F1270m and cr_alr2535R226F, a fragment of 1523 bp can be amplified from WT, but no amplification from *ΔnatI* as cr_alr2535R226F was located in the deleted part. In 5, primers Pall1284F1359m and P190R are used, leading to a PCR fragment of 1597 bp in WT but no amplification from *ΔnatD*. In 6, primers P281F and P982R are used, with a PCR fragment of 702 bp from the WT but no amplification from *ΔbgtB*.

Supplemental Experimental procedures

Construction of Plasmids, Mutants

All mutants were generated by conjugation followed by either conventional homologous recombination [7], or genome editing technique based on Cpf1 [5,6]. The strains are listed in Table S3, and all the oligonucleotides are presented in Table S4.

All plasmids were verified by DNA sequencing. The plasmids pCint2-Mall1046, pCint2-Malr4167 and pCint2-Malr3187 that carried *sacB*, based on the integrative vector pCint2 [4], were used to obtain the mutants *ΔnatA*, *ΔbgtA* and *ΔbgtB*, respectively, in which a large part of the coding region was replaced by antibiotic-resistance marker (for details, see Figure S1). To construct these plasmids, the vector pCint2 was amplified by primers PV_14 and PV_13, and the neomycin-resistance gene cassette was amplified by PtstspF and Pgfp_spR from plasmid pSfgfp-npt that carried nptII gene from pRL25Z [8]. The upstream fragments for the three genes used for homologous recombination were amplified by primer pairs Pall1046F1256m/Pall1046R120, Palr4167F1081m/Palr4167R261 and Palr3187F1030m/Palr3187R264, respectively, using the genomic DNA of *Anabaena* as template. Similarly, their downstream DNA arms for

homologous recombination were amplified, respectively by using primer pairs Pall1046F721/Pall1046R2119, Palr4167F697/Palr4167R2200 and Palr3187F1042/Palr3187R2328. After PCR, the fragments corresponding to the vector, the resistance marker, the upstream and downstream fragments were ligated by Multis one step cloning system. The final constructs were named as pCint2-Mall1046, pCint2-Malr4167 and pCint2-Malr3187, for the inactivation of *natA*, *bgtA*, *bgtB*, respectively. An additional construct, pCint2-Malr4167-sp, was also obtained using the same approach; it is similar to pCint2-Malr4167 but carries a spectinomycin-resistance marker.

The plasmids pCpf1-Malr4165R126, pCpf1-Malr2535R226 and pCpf1-Mall1284F610 were used to construct markerless mutants *ΔnatG*, *ΔnatI* and *ΔnatD* respectively through Cpf1 genome editing system [6]. To construct pCpf1-Malr4165R126 and pCpf1-Malr2535R226, the vector pCpf1 was linearized by restriction enzyme BglII and BamHI [5]. The upstream fragments for Cpf1-based homologous recombination were amplified by primer pairs Palr4165F1188m/Palr4165R45 and Palr2535F1270m/Palr2535R45, respectively, from the genomic DNA of *Anabaena*. The downstream fragments for Cpf1-based homologous recombination were amplified by primer pairs Palr4165F898/Palr4165R2078 and Palr2535F763/Palr2535R1849, respectively, from the genomic DNA of *Anabaena*. Then the vector, the upstream and downstream fragments were ligated by ClonExpress MultiS One Step Cloning Kit (Vazyme Biotech Co.,Ltd; Nanjing, China) to construct the precursor plasmids. Single-stranded oligonucleotide pairs cr_alr4165R126F/cr_alr4165R126R and cr_alr2535R226F/cr_alr2535R226R were heated to 94 ℃ for 2 min and annealed following cooling to form double-stranded oligonucleotides to be used as guide sequences [5], respectively. To complete the construction of pCpf1-Malr4165R126 and pCpf1-Malr2535R226, the precursor plasmids were digested by AarI then the corresponding guide sequence was inserted through T4 ligase. To construct pCpf1-Mall1284F610, the vector pCpf1 was digested by AarI and the double-stranded oligonucleotides (cr_all1284F610F/cr_all1284F610R) as guide sequence was inserted to construct the precursor plasmid. To complete the construction of pCpf1-Mall1284F610, we linearized the precursor plasmid by restriction enzyme BglII and BamHI. Then the linearized precursor plasmid and the upstream and downstream fragments for homologous recombination amplified by primers Pall1284F1359m/Pall1284R6 and Pall1284F865/Pall1284R2168 respectively were ligated by ClonExpress MultiS One Step Cloning Kit.

To construct *ΔnatA*, *ΔbgtA* and *ΔbgtB*, the plasmids pCint2-Mall1046, pCint2-Malr4167 and pCint2-Malr3187 were introduced, respectively, into *Anabaena* by conjugation through triparental mating, as described [9]. The double mutant *ΔnatAΔbgtA* was obtained by transferring the plasmid pCint2-Malr4167-sp into the *ΔnatA* mutant. To construct *ΔnatG*, *ΔnatI* and *ΔnatD*, the plasmids pCpf1-Malr4165R126, pCpf1-Malr2535R226 and pCpf1-Mall1284F610 were introduced into *Anabaena* by conjugation, followed the procedure as described for Cpf1-based genomic editing [6]. All mutants were confirmed by PCR.

Genomic Sequencing, Assembly and Comparison

The whole genome sequencing was performed by the BGI Company, using the second-generation sequencing technique, with coverage at about 97–99% [10]. Whole genome comparison was done by BGI using MUMmer [11]. Briefly, the genomes of the indicated strains were sequenced using an Illumina HiSeq 4000 system (Illumine, San Diego, CA, USA). Genomic DNA was sheared randomly to construct three read libraries by a Bioruptor ultrasonicator (Diagenode, Denville, NJ, USA) and physic-chemical methods. The paired-end fragment libraries were sequenced according to the Illumina Hiseq 4000 system's protocol. Raw reads of low quality from paired-end sequencing (those with consecutive bases covered by fewer than five reads) were discarded. The sequenced reads were assembled using SOAP de novo v1.05 software (BGI Company, Shenzhen, China).

References

1. Pernil, R.; Picossi, S.; Mariscal, V.; Herrero, A.; Flores, E. ABC-type amino acid uptake transporters Bgt and N-II of

Anabaena sp. strain PCC 7120 share an ATPase subunit and are expressed in vegetative cells and heterocysts. *Mol. Microbiol.* **2008**, *67*, 1067–1080.

2. Pernil, R.; Picossi, S.; Herrero, A.; Flores, E.; Mariscal, V. Amino Acid Transporters and Release of Hydrophobic Amino Acids in the Heterocyst-Forming Cyanobacterium *Anabaena* sp. Strain PCC 7120. *Life (Basel)* **2015**, *5*, 1282–1300.
3. Picossi, S.; Montesinos, M.L.; Pernil, R.; Lichtle, C.; Herrero, A.; Flores, E. ABC-type neutral amino acid permease N-I is required for optimal diazotrophic growth and is repressed in the heterocysts of *Anabaen*a sp. strain PCC 7120. *Mol. Microbiol.* **2005**, *57*, 1582–1592.
4. Zhang, J.Y.; Lin, G.M.; Xing, W.Y.; Zhang, C.C. Diversity of Growth Patterns Probed in Live Cyanobacterial Cells Using a Fluorescent Analog of a Peptidoglycan Precursor. *Front. Microbiol.* **2018**, *9*, 791.
5. Niu, T.C.; Lin, G.M.; Xie, L.R.; Wang, Z.Q.; Xing, W.Y.; Zhang, J.Y.; Zhang, C.C. Expanding the potential of CRISPR-Cpf1 based genome editing technology in the cyanobacterium *Anabaena* PCC 7120. *ACS Synth. Biol.* **2018**, *8*, 170–180
6. Ungerer, J.; Pakrasi, H.B. Cpf1 Is A Versatile Tool for CRISPR Genome Editing Across Diverse Species of Cyanobacteria. *Sci. Rep.* **2016**, *6*, 1–9.
7. Cai, Y.P.; Wolk, C.P. Use of a conditionally lethal gene in *Anabaena* sp. strain PCC 7120 to select for double recombinants and to entrap insertion sequences. *J. Bacteriol.* **1990**, *172*, 3138–3145.
8. Zhang, J.Y.; Chen, W.L.; Zhang, C.C. *hetR* and *patS*, two genes necessary for heterocyst pattern formation, are widespread in filamentous nonheterocyst-forming cyanobacteria. *Microbiology* **2009**, *155*, 1418–1426.
9. Elhai, J.; Wolk, C.P. Conjugal transfer of DNA to cyanobacteria. *Methods Enzymol.* **1988**, *167*, 747–754.
10. Li, R.; Li, Y.; Fang, X.; Yang, H.; Wang, J.; Kristiansen, K.; Wang, J. SNP detection for massively parallel whole-genome resequencing. *Genome. Res.* **2009**, *19*, 1124–1132.
11. Kurtz, S.; Phillippy, A.; Delcher, A.L.; Smoot, M.; Shumway, M.; Antonescu, C.; Salzberg, S.L. Versatile and open software for comparing large genomes. *Genome. Biol.* **2004**, *5*, R12.
